# Dietary intake of nutrients and its correlation with fatigue in multiple sclerosis patients

**Published:** 2014

**Authors:** Sama Bitarafan, Mohammad-Hossein Harirchian, Shahriar Nafissi, Mohammad-Ali Sahraian, Mansoureh Togha, Fereydoun Siassi, Ahmad Saedisomeolia, Elham Alipour, Nakisa Mohammadpour, Maryam Chamary, Niyaz Mohammadzadeh Honarvar, Ali-Akbar Saboor-Yaraghi

**Affiliations:** 1 Department of Cellular and Molecular Nutrition, School of Nutritional Sciences and Dietetics, Tehran University of Medical Sciences, Tehran, Iran; 2 Department of Neurology AND Iranian Center of Neurological Research, Imam Khomeini Hospital, Tehran University of Medical Sciences, Tehran, Iran; 3 Department of Neurology AND Shariati Hospital, Tehran University of Medical Sciences, Tehran, Iran; 4 Department of Neurology, Sina MS Research Center, Tehran University of Medical Sciences, Tehran, Iran; 5 Department of Community Nutrition, Tehran University of Medical Sciences, Tehran, Iran

**Keywords:** Dietary Intake, Folate, Magnesium, Modified Fatigue Impact Scale, Multiple Sclerosis

## Abstract

**Background:**

The role of nutrition in the progression of multiple sclerosis (MS) and related complications such as fatigue has been reported by several studies. The aim of this study is the assessment of nutritional status and its relationship with fatigue in multiple sclerosis patients.

**Methods:**

This is a cross-sectional study, in which 101 relapsing-remitting MS patients were enrolled. The fatigue status was determined using the validated Persian version of of the Modified Fatigue Impact Scale (MFIS). Dietary intake was assessed using a 3-day food record questionnaire and compared to dietary reference intake (DRI) values. Association between variables was determined using Pearson Correlation Coefficient.

**Results:**

In the preset study, 25 men and 76 women (total = 101) were enrolled. Analysis of dietary intake showed that daily intake of vitamin D, folate, calcium, and magnesium were significantly lower than DRI in all of patients. In men, zinc intake was significantly lower than DRI; while, in women, iron was significantly below the DRI level. After adjusting for energy, MFIS and its physical subscale were highly correlated with intake of folate and magnesium.

**Conclusion:**

Our findings support that lower magnesium and folate diets are correlated with higher fatigue scores in MS patients.

## Introduction

Multiple sclerosis is a chronic inflammatory disease of the central nervous system. Environmental factors such as diet have a fundamental role in the pathogenesis and progression of MS.^1–3^ The relationship between MS and nutrition has been investigated by several investigators; however, the nutritional status has not been studied among MS patients.^4^

Fatigue is one of the most common complications among MS patients.^5^ Although the pathophysiology of fatigue is not yet clear in this population, inflammatory cytokines, axonal atrophy, hypometabolism of specific brain regions, and higher energy requirement are recognized as probable etiologic factors.^6–9^ Nutritional status may affect MS complications such as fatigue.^10^ Fatigue can be worsened by an imbalanced diet.^4^ Deficiency of some nutrients such as folate and magnesium have been reported in chronic fatigue syndrome (CFS) patients.^11, 12^

One of the most applicable instruments for fatigue evaluation is MFIS; it measures the fatigue impact on physical, cognitive, and psychosocial functioning.^13–14^ The DRI is an indicator that determines the estimated average requirement of nutrients, as the median population intake needs, and calculates the recommended dietary allowance (RDA). RDA is the level of nutrient intake that is sufficient to cover 97.5% of the population's requirement and determines with 2 standard deviations different from median need.^15^

Currently, there are no data on the effect of nutritional status of MS patients on their fatigue status. The aim of this study was to assess the dietary intake of different nutrients in MS patients in comparison with DRI values and its correlation with their fatigue status.

## Materials and Methods

### Enrolment of subjects

In the present study, 101 relapsing remitting multiple sclerosis (RRMS) patients (according to the McDonald criteria) were enrolled between 2010 and 2012 in the Iranian Center of Neurological Research, Imam Khomeini Hospital, Tehran, Iran.^16^ The age of the enrolled population ranged from 20 to 40 years, and their Expanded Disability Status Scale (EDSS) scores from 0 to 4.5. Body mass index (BMI) of patients was lower than 30. Patients were not in the acute phase of the disease and had not relapsed for at least 3 months preceding the study. There was no report of any nutritional supplement intake during the recent 3 months or any medication except weekly interferon beta-1a injection. Patients with alcohol intake, any possible addiction, dysphagia, diabetes, inflammatory bowel disease, and liver, pancreatic, and biliary disorders were excluded from the study.

### Clinical characteristics

Age, gender, and anthropometric measurements of all patients were recorded. The survey applied the validated Persian Modified Fatigue Impact Scale self-assessment questionnaires.^17–18^ The items of MFIS were divided into three subscales of physical, cognitive, and psychosocial scores.^5^ It can also be computed as a total score (ranges from 0-84). The higher scores indicate the greater fatigue status in patients. Neurologic impairment of the patients was assessed by a neurologist using the Kurtzke EDSS.^19^ The patients completed a 24-hour food record for 3 days and then all diaries were checked by a dietitian.

### Statistical analysis

Nutritional data were analyzed using Nutritionist IV software (San Bruno, CA, USA, First Data Bank). Statistical analysis was undertaken using SPSS for Windows (version 18; SPSS Inc., Chicago, IL, USA). Student's independent t-test was employed to compare nutrient intake with DRI values (2012). Association between variables was determined using Pearson Correlation Coefficient. Partial correlation was employed for controlling of daily energy intake. P < 0.05 were considered statistically significant.

## Results

In the present study, the study population consisted of 101 RRMS patients with mean EDSS of 1.02 ± 0.99, including 25 men (aged 30.24 ± 7.37) and 76 women (aged 31.82 ± 5.96) (Table 1). Mean BMI of our patients was 24.21 ± 3.79 kg/m^2^ (Table 1).

**Table 1:** General characteristics of subjects

General characteristics	Mean ± SD
Age (year): Female (n = 76) Male (n = 25)	31.82 ± 5.96 30.24 ± 7.37
Weight (kg)	66.11 ± 12.19
Height (meter)	1.65 ± 0.08
BMI (kg/m^2^)	24.21 ± 3.79
EDSS	1.02 ± 0.99

BMI: Body mass index; EDSS: Expanded disability status scale

Mean energy intake was 1965.56 ± 320.98 kcal/day. The intake of carbohydrates, proteins, and fats, respectively, constitute 56.10%, 12.20%, and 31.70% of total daily energy intake. Daily fat intake consists of three subgroups of saturated fatty acid (SFA) that was 8.82% of the daily energy intake, monounsaturated fatty acid (MUFA) that was 12.24% of the daily energy intake, and polyunsaturated fatty acid (PUFA) that was 10.64% of the daily energy intake (data not presented). Mean fiber intake was 14.17 ± 3.71 grams in men, and 14.69 ± 4.59 grams in women; they were significantly lower than DRI values (Table 2).

**Table 2:** Mean daily nutrient intake in MS patients and their significant differences with DRI values

Nutrients	Intake of males (Mean ± SD)	DRI for males	P	Intake of females (Mean ± SD)	DRI for females	P
Vitamin D (µg)	2.77 ± 10.77	15	< 0.001	2.14 ± 12.59	15	< 0.001
Folate (µg)	268.41 ± 66.93	400	< 0.001	284.15 ± 81.17	400	< 0.001
Iron (mg)	12.33 ± 3.47	8	< 0.001	11.79 ± 3.54	18	< 0.001
Zinc (mg)	8.22 ± 2.50	11	< 0.001	7.66 ± 1.90	8	0.134
Calcium (mg)	631.36 ± 232.29	1000	< 0.001	583.99 ± 305.50	1000	< 0.001
Magnesium (mg)	242.24 ± 63.97	420	< 0.001	240.25 ± 57.90	320	< 0.001
Fiber (g)	14.17 ± 3.71	38	< 0.001	14.69 ± 4.59	25	< 0.001

Daily dietary intake of vitamin D, folate, calcium, and magnesium were significantly lower than DRI (Table 2). In men, zinc was significantly lower and iron was higher than DRI. However, in women iron was significantly below DRI levels (Table 2).

After adjusting for daily energy intake, there were significant correlations between increasing total of MFIS, and decreasing daily intake of folate (P = 0.02) and magnesium (P = 0.03) (Table 3). This correlation was significant between physical subscale of MFIS and daily intake of folate (P = 0.008) and magnesium (P = 0.003). MFIS scores were not significantly associated with vitamin D and calcium intake, and intake of any nutrients in the present study (Table 3).

**Table 3:** Correlation between MFIS scores and deficient nutrients

MFIS	Scores (Mean ± SD)	P			

		Folate	Magnesium	Vitamin D	Calcium
Physical	12.97 ± 8.34	0.008*	0.003*	0.63	0.22
Cognitive	12.96 ± 9.52	0.15	0.21	0.86	0.06
Psychological	2.20 ± 2.31	0.26	0.35	0.82	0.93
Total	28.13 ± 17.93	0.02*	0.03*	0.77	0.11

* P < 0.05: Significant relationship

## Discussion

In the present study, for the first time, dietary intake of all nutrients (macronutrients and micronutrients), in comparison with DRI values, were assessed in RRMS patients (separately in men and women). Moreover, correlations between daily intake of all nutrients and fatigue scores were evaluated.

DRI values are the minimum amount of each nutrient's intake that is necessary for healthy persons. Thus, DRI may be different for individuals with specific diseases such as MS.^15^ In MS patients, during disease progression, many nutrients’ requirements, such as zinc and iron, are increased.^20^ Therefore, in this study DRI was used instead of a control group for assesment of nutritional status of MS patients. Some researchers, such as Timmerman and Stuifbergin, and Saka et al., also used DRI instead of a control group for diet evaluation in MS patients.^21–22^

Considering that the disability of MS patients affect their dietary intake and obesity augments MS patients disability and fatigue, in this study many confounder factors were controlled by inclusion criteria.^4, 21, 23^

These factors are as follows:

- Severity of disability: Mean EDSS of patients was 1.02 ± 0.99.
- Obesity: All patients’ BMI were < 30.
- The course of disease: All patients were RRMS.
- Supplementation: No patients used any supplements.
- Male to female ratio: This ratio obtained 1/3 that was representative of MS prevalence in the Iranian population.^24^

The results obtained in this study were discussed in two ways:

- Nutritional status in MS patients and deficiency of nutrients
- Relationship among nutritional intake deficiencies and fatigue scores in MS patients

Results of this study showed that mean daily energy intake (1965.56 ± 320.98 kcal/day) and percentage of total daily energy provided by carbohydrates (56.1%), proteins (12.2%), and fats (31.7%) were in accordance with DRI. These findings are in line with the study of Saka et al. that similarly showed the percentage of total energy of the carbohydrates (46.9%), proteins (14.6%), and fats (38.4%) were approximately according to DRI^22^

In this study, male and female patients received 37.28% and 58.76% of the daily recommendations for fiber, which confirms results of the studies by Hewson et al. and Timmerman and Stuifbergin.^21, 23^ In our study, daily intake of MUFA (12.24% of energy) and PUFA (10.64% of energy) were in accordance with DRI. Another study showed that MS patients and healthy subjects receive similar amounts of different fatty acids.^25^

Among micronutrients the daily intake of which were measured, followed nutrients intake were lower than DRI values in the diet of MS patients.

Dietary intake of zinc among male patients was lower than DRI. The findings of the study of Ramsaransing et al. indicated that dietary intake of zinc was lower than DRI in MS patients regardless of their gender.^20^

Dietary intake of iron among female patients was lower than DRI; however, iron intake was higher than DRI among male patients. Timmerman and Stuifbergin found that women with MS had an iron consumption higher than that recommended; this result was not in agreement with our findings.^21^

We found that daily calcium intake was lower than DRI among both male and female patients. This finding is in accordance with the study of Timmerman and Stuifbergin that surveyed the nutritional status in women with MS and found that calcium intake is lower than recommended.^21^ Because of the high saturated fat content of dairy products, MS patients are commonly advised to refrain from eating these products. This may explain their lower calcium intake. Not only is it not clear whether the lower intake of dairy products can improve the disease progression, but also Ramsaransing et al. showed that low calcium and iron intake may be correlated with progression of MS.^4, 20^

Daily intakes of magnesium and folate were lower than DRI values in our study. These results confirmed the findings of Ramsaransing et al. that showed a lower intake of magnesium and folate in MS patients in comparison to DRI.^20^

Vitamin D intake of our MS patients was significantly lower than DRI values. Several studies have shown that most MS patients are vitamin D deficient in comparison with the healthy population.^4^ Due to immunoregulatory properties, vitamin D deficiency is considered as a risk factor for MS.^26^

Higher prevalence of fatigue among MS patients is a major concern and is defined as a subjective lack of mental or physical energy.^7, 27–28^ In the present study, no daily nutrient's intake, except magnesium and folate, was correlated with fatigue scores. We found that lower dietary intake of magnesium and folate in MS patients is correlated with higher score of MFIS and its physical subscale.

As previously shown, deficiency of magnesium can be a risk factor for MS.^29^ It has been reported that red blood cells’ magnesium levels in chronic fatigue syndrome patients were lower than normal values. It is interesting that the symptoms of CFS and magnesium deficiency are similar.^11^ In addition, magnesium supplementation can improve the symptoms of CFS when deficiency of this nutrient is presented.^12^ Yasui et al. found the concentration of magnesium in the central nervous system of MS patients to be lower than controls.^30^

Mechanism of exacerbation of MS symptoms in magnesium deficiency is probably the reduction of enzyme inhibition of nitric oxide synthase and production of more nitric oxide in macrophages.^20^ Magnesium deficiency can also induce inflammatory cytokines production.^31^

Folate is considered as an important nutrient in neurodegenerative diseases.^32–33^ Moreover, folic acid levels are reduced in cerebrospinal fluid of many CFS patients. Chronic reduction of serum and then cerebrospinal fluid folate level, results in lower brain folate content that may impair brain function and induce CFS. Godfrey et al. and Jacobson et al. found that folate supplementation showed positive effects on CFS improvement.^34–35^ The mechanisms of folate action is not fully understood; however, this molecule can reduce the circulatory level of homocysteine, a cytotoxic amino acid for neural cells.^32^

This is a preliminary study and may provide some direction for further researches. For further researches, we suggest measuring serum level of nutrients, using food frequency questionnaire (FFQ) besides food records, and designing clinical trials.

We did not measure the serum levels of the studied nutrients. Furthermore, we did not compare dietary intake of MS patients with a healthy control group. These are the limitations of the present study. Therefore, we interpret our data cautiously due to this limitation and only emphasize the significant association among low folate and magnesium diets and increasing of fatigue score in MS patients. Assessment and improvement of nutritional status of MS patients should account as a complementary approach to their usual treatments.^3–4^ The evaluation of the nutritional status of patient with MS and designing a program for correction of nutrient deficiencies according to DRI levels, are strongly recommended. This may be effective in MS patients achieving a healthy life and improving their fatigue status.

## Conclusion

The results of nutritional status analysis of 101 RRMS patients showed that dietary intakes of vitamin D, folate, calcium, and magnesium were lower than DRI. Lower magnesium and folate diets were correlated with higher fatigue scores in these patients. To our knowledge, there is no nutritional recommendation as complementary therapy in treatment of fatigue related to multiple sclerosis. Therefore, as a preliminary study this result can be helpful. We suggest recognizing and correcting the low dietary intake of nutrients in MS patients, according to DRI values, with specific consideration to folate and magnesium. This may improve fatigue syndrome in MS patients.
